# Early administration of abciximab reduces mortality in female patients with ST-elevation myocardial infarction undergoing primary percutaneous coronary intervention (from the EUROTRANSFER Registry)

**DOI:** 10.1007/s11239-012-0826-3

**Published:** 2012-10-14

**Authors:** Artur Dziewierz, Zbigniew Siudak, Tomasz Rakowski, Paweł Kleczyński, Jacek S. Dubiel, Dariusz Dudek

**Affiliations:** 1 2nd Department of Cardiology, Jagiellonian University Medical College, 17 Kopernika Street, 31-501 Krakow, Poland; 2 Department of Interventional Cardiology, Jagiellonian University Medical College, 17 Kopernika Street, 31-501 Krakow, Poland

**Keywords:** Gender, Female, Myocardial infarction, Abciximab, Angioplasty

## Abstract

The present study assessed the impact of early administration of abciximab in female and male patients with ST-segment elevation myocardial infarction (STEMI) transferred for primary angioplasty (PPCI). Data were gathered for 1,650 consecutive patients with STEMI transferred for PPCI from hospital networks in seven countries in Europe from November 2005 to January 2007 (the EUROTRANSFER Registry population). Among 1,086 patients who received abciximab, there were 186 women and 541 men who received abciximab early (>30 min before PPCI), and 86 women and 273 men treated with late abciximab. Female patients were high-risk individuals, with advanced age and increased rate of ischemic events. Early abciximab administration was associated with enhanced patency of the infarct-related artery before PPCI, and improved epicardial flow after PPCI in both women and men. Early abciximab in women led to the decrease in ischemic events, including 30 day (adjusted OR 0.26, 95 % CI 0.10–0.69, *p* = 0.007) and 1 year (adjusted OR 0.37, 95 % CI 0.16–0.84, *p* = 0.017) mortality reduction. In contrast, the reduction in 30 day (adjusted OR 0.69, 95 % CI 0.35–1.39, *p* = 0.27) and 1 year (adjusted OR 0.68, 95 % CI 0.38–1.22, *p* = 0.19) mortality was not significant in men. The frequency of bleeding events was similar in the early abciximab group compared to the late abciximab group in both women and men. Early administration of abciximab improved patency of the infarct-related artery before and after PPCI, and led to improved survival in female patients with STEMI.

The mortality benefit of glycoprotein (GP) IIb–IIIa inhibition during primary percutaneous coronary intervention (PCI) for ST-segment elevation myocardial infarction (STEMI) is limited to high-risk patients [1]. An additional favorable effect in that high-risk group of patients may be achieved when GPIIb–IIIa inhibitor (abciximab) is given early, before primary PCI for STEMI [2–6]. Whether the advantages of upstream abciximab are independent of gender, an important determinant of outcomes after primary PCI, has not been reported.

The present study assessed the impact of early administration of abciximab in female and male patients with STEMI transferred for primary PCI, based on data from the European Registry on patients with ST-elevation MI transferred for mechanical reperfusion with a special focus on upstream use of abciximab (EUROTRANSFER) [2–4].

## Methods

The EUROTRANSFER Registry (http://ClinicalTrials.gov, number NCT00378391) design and main results have been previously published [2–4]. In this registry data on 1,650 consecutive patients with STEMI transferred for primary PCI in 15 STEMI hospital networks from seven European countries from November 2005 to January 2007 were collected. To assess the impact of early administration of abciximab on clinical outcomes of women and men data on 1,086 patients who received abciximab were retrieved from the registry database. Patients were stratified based on gender (women vs men) and abciximab administration strategy (early vs late administration). Patients who received abciximab before or during transfer to the PCI-hospital, at least 30 min before first balloon inflation, or coronary angiography in patients who did not undergo PCI were classified as early abciximab patients. The remaining patients, in whom abciximab was given <30 min to or during PCI, were analyzed as late abciximab group. Thrombolysis In Myocardial Infarction (TIMI) risk score for STEMI was calculated for each patient as described previously [2, 7]. Patients with ≥3 points in TIMI risk score on admission were considered as “high-risk” patients. The study protocol and execution complied with the declaration of Helsinki and was approved by the institutional review board.

The primary end point of the present analysis was 1 year all-cause mortality. In addition, rates of all-cause death, nonfatal reinfection, urgent revascularization (PCI or coronary artery bypass grafting), puncture site hematoma, intracranial hemorrhage, and major bleeding requiring transfusion at 30 days after primary PCI were assessed [2, 3]. TIMI flow in the infarct-related artery before and after primary PCI, ST-segment resolution after PCI, and rate of angiographic PCI complications (no-reflow, distal embolization) were assessed by visual estimation of local investigators.

Results were presented as percentages of patients or medians (interquartile ranges) where applicable. Differences between groups stratified by gender and abciximab administration strategy were assessed using Chi-square test and Fisher’s exact test for dichotomous variables and Mann–Whitney *U* test for continuous variables. The effect of receiving early abciximab versus late abciximab on clinical outcome parameters was presented as odds ratio and 95 % confidence interval. To adjust for possible selection bias, propensity score [8] for the likelihood of receiving abciximab early was calculated based on the following variables: sex, age, body mass index, medical history (previous myocardial infarction, chronic kidney disease, previous heart failure symptoms, previous PCI, previous coronary artery bypass grafting, previous stroke, smoking status, diabetes mellitus, peripheral arterial disease), medications before admission (clopidogrel and thrombolysis before PCI-hospital), time from chest pain onset to diagnosis, diagnosis to balloon time and the location where the STEMI diagnosis was made (ambulance or referral hospital). Differences in clinical outcomes between patients treated with early abciximab versus late abciximab were adjusted for propensity score using logistic regression analysis and presented as adjusted odds ratio with 95 % confidence interval. Difference in death rates between groups during follow-up was assessed by the Kaplan–Meier method using log-rank test. All tests were 2-tailed and a *p* value <0.05 was considered statistically significant. All analyses were performed with SPSS 15.0 (SPSS Inc., Chicago, Illinois).

## Results

Data on 1,650 patients were entered into the EUROTRANSFER Registry. Overall, 1,086 patients, including 272 women (25 %) and 814 men receiving abciximab were identified (study group). Among women 186 patients (68.4 %) were classified as early abciximab patients, and 86 as late abciximab patients. Similarly, among men 541 patients (66.5 %) were classified as early abciximab patients, and 273 as late abciximab patients (*p* = 0.56).

Women were older (women vs men, median age: 70.5 (62–79) vs 62 (52–71) years, *p* < 0.0001), with a higher prevalence of diabetes mellitus (19.5 vs 14.1 %, *p* = 0.034), and have experienced longer delays from symptom onset to PCI (median time: 226 (160–335) vs 206 (143–321) min, *p* = 0.19) than men. In addition, the percentage of patients classified as “high-risk” (TIMI risk score ≥3 points) was higher in women than men (74.3 vs 50.9 %, *p* < 0.001). Clinical, angiographic and procedural characteristics of the study patients according to gender and abciximab administration strategy are shown in Table 1. Baseline characteristics were similar in the early abciximab group compared to the late abciximab group in both women and men, except more advanced age and less frequent chronic kidney disease presence in male patients receiving abciximab early than late. As shown in Table 1, women receiving abciximab late were more likely to be treated with drug-eluting stents during primary PCI than women receiving abciximab early. Femoral access site and thrombus aspiration catheters were less frequently used in male patients receiving abciximab early than late.

**Table 1:** Clinical, angiographic and procedural characteristics of women and men stratified by abciximab administration strategy

Variable	Women (*n* = 272)		*p* value	Men (*n* = 814)		*p* value
	Early abciximab (*n* = 186)	Late abciximab (*n* = 86)		Early abciximab (*n* = 541)	Late abciximab (*n* = 273)	
Age (years)	69 (60–78)	73 (65–79)	0.15	63 (52–71)	60 (51–71)	0.038
Age ≥75 years	39.2 %	43.0 %	0.60	16.1 %	12.8 %	0.25
Body mass index (kg/m^2^)	26.6 (24.0–29.4)	26.3 (23.2–31.1)	0.59	26.8 (24.3–29.3)	26.8 (24.4–29.7)	0.64
Previous myocardial infarction	8.6 %	11.6 %	0.51	10.9 %	13.6 %	0.30
Previous heart failure symptoms	0.5 %	2.3 %	0.24	0.9 %	0.7 %	0.99
Previous percutaneous coronary intervention	7.0 %	11.6 %	0.20	7.6 %	9.5 %	0.35
Previous coronary artery bypass grafting	0.5 %	2.3 %	0.24	1.1 %	2.2 %	0.23
Current smoker	25.3 %	29.1 %	0.55	38.6 %	40.3 %	0.65
Diabetes mellitus	21.0 %	16.3 %	0.41	13.5 %	15.4 %	0.52
Insulin	9.1 %	5.8 %	0.48	3.7 %	4.0 %	0.85
Peripheral arterial disease	2.7 %	3.5 %	0.71	2.4 %	2.9 %	0.82
Previous stroke	4.3 %	2.3 %	0.51	3.5 %	2.9 %	0.69
Chronic kidney disease	2.2 %	2.3 %	0.99	1.5 %	4.0 %	0.028
Time from symptoms onset to diagnosis (min)	110 (65–200)	121 (61–235)	0.64	85 (50–180)	100 (60–196)	0.13
Aspirin before cathlab	94.6 %	88.4 %	0.08	95.9 %	92.7 %	0.06
Clopidogrel before cathlab	21.5 %	25.6 %	0.53	21.4 %	26.7 %	0.10
Unfractionated heparin before cathlab	77.4 %	69.8 %	0.23	69.7 %	68.5 %	0.75
Heart rate on admission (beats per min)	74 (65–88)	80 (66–90)	0.32	75 (66–86)	80 (68–90)	0.06
Systolic blood pressure on admission (mmHg)	130 (110–150)	138 (110–160)	0.45	130 (117–150)	134 (114–149)	0.55
Diastolic blood pressure on admission (mmHg)	76 (65–90)	80 (66–90)	0.67	80 (70–90)	80 (70–90)	0.08
Killip class IV on admission	2.7 %	3.5 %	0.71	2.6 %	4.0 %	0.29
TIMI risk score ≥3 points	71.5 %	80.2 %	0.14	51.8 %	49.1 %	0.50
Femoral access	83.3 %	90.7 %	0.14	81.5 %	89.0 %	0.006
LAD as infarct-related artery	44.6 %	43.0 %	0.90	48.4 %	45.4 %	0.46
Multivessel disease	49.7 %	52.3 %	0.70	49.2 %	51.5 %	0.55
Intra-aortic balloon pumping	3.2 %	4.7 %	0.73	3.9 %	4.4 %	0.85
Immediate PCI	*n* = 175	*n* = 80		*n* = 516	*n* = 256	
Time from symptoms onset to PCI (min)	223 (165–329)	237 (145–359)	0.98	201 (140–309)	213 (153–340)	0.15
Stents	86.3 %	87.5 %	0.85	94.2 %	96.9 %	0.11
Drug-eluting stent	24.5 %	38.6 %	0.038	33.5 %	35.5 %	0.62
Direct stenting	15.2 %	17.1 %	0.84	18.1 %	19.8 %	0.62
Thrombus aspiration	10.3 %	11.2 %	0.83	9.7 %	19.5 %	<0.001
Non-infarct-related artery PCI	4.6 %	5.0 %	0.99	5.6 %	4.7 %	0.62

Values are presented as percentages or medians (interquartile ranges)

*LAD* left anterior descending artery, *PCI* percutaneous coronary intervention, *TIMI* thrombolysis in myocardial infarction

**Fig. 1:**
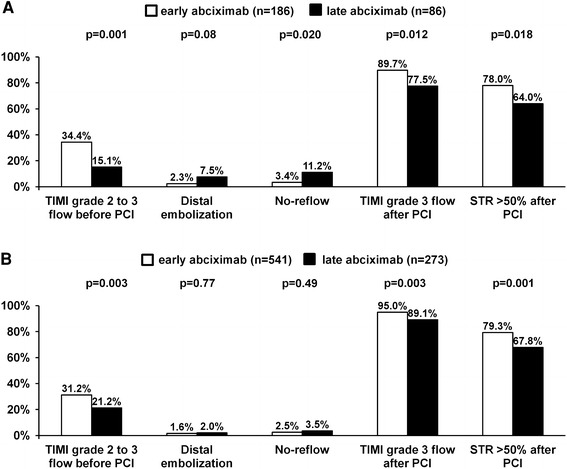
Angiographic and electrocardiographic characteristics of women (**a**) and men (**b**) stratified by abciximab administration strategy (early abciximab-*empty bars* late abciximab *solid bars*). *PCI* percutaneous coronary intervention, *STR* ST-segment resolution, *TIMI* thrombolysis in myocardial infarction

Despite no difference in frequency of initial TIMI grade 2–3 flow between women and men (women vs men: 28.3 vs 27.9 %, *p* = 0.89), the rates for final TIMI grade 3 flow after PCI (85.9 vs 93.0 %, *p* < 0.001) were lower in female patients. In addition, women were at higher risk of no-reflow (5.9 vs 2.8 %, *p* = 0.024) and distal embolization (3.9 vs 1.7 %, *p* = 0.036) during primary PCI. However, the rates for ST-segment resolution >50 % assessed 60 min after PCI were similar in both groups (73.5 vs 75.4 %, *p* = 0.53). Early administration of abciximab before primary PCI was associated with enhanced patency of the infarct-related artery before PCI, and improved epicardial flow, as well as ST-segment resolution after PCI (Fig. 1) in both women and men. In contrary, benefits of early use of abciximab in terms of the reduction in angiographic complications of PCI were limited to female patients.

**Fig. 2:**
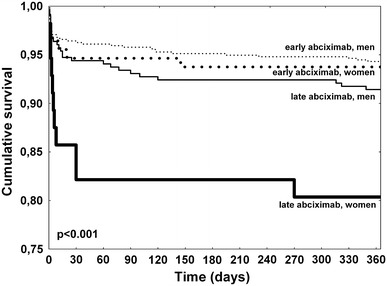
Kaplan–Meier survival curves stratified by gender and abciximab administration strategy

Higher rates for all bleeding events (women vs men: 15.1 vs 7.7 %, *p* < 0.001), as well as 30 day (7.7 vs 4.3 %, *p* = 0.027) and 1 year (10.7 vs 6.1 %, *p* = 0.013) death were observed in female patients. Strategy of early initiation of abciximab in women led to significant decrease in ischemic events including 30 day and 1 year mortality reduction. Importantly, observed difference was significant after adjustment for potential confounders (Table 2). In contrary, among men only a trend towards improved 1 year survival related to early treatment with abciximab was confirmed. The frequency of bleeding events in the early abciximab group was similar to that observed in the late abciximab group in both women and men (Table 2). As shown in the Fig. 2, the 1 year survival among women receiving early abciximab was as good as for men receiving abciximab either early or late.

**Table 2:** Clinical outcomes of women and men stratified by abciximab administration strategy

Variable	Early abciximab	Late abciximab	OR (95 % CI)	*p* value	Adjusted OR (95 % CI)	*p* value
Women	*n* = 186	*n* = 86				
30 day						
Death	4.8 %	14.0 %	0.31 (0.13–0.78)	0.023	0.26 (0.10–0.69)	0.007
Death + nonfatal reinfarction	6.5 %	16.3 %	0.36 (0.16–0.80)	0.013	0.29 (0.12–0.69)	0.005
Death + nonfatal reinfarction + urgent revascularization	7.5 %	17.4 %	0.39 (0.18–0.84)	0.016	0.32 (0.14–0.74)	0.007
Major bleeding requiring transfusion	5.4 %	2.3 %	2.39 (0.51–11.13)	0.27	2.22 (0.47–10.57)	0.32
Intracranial haemorrhage	0 %	0 %	–	–	–	–
Puncture site haematoma	12.9 %	9.3 %	1.44 (0.62–3.36)	0.39	1.21 (0.51–2.88)	0.67
All bleeding	16.7 %	11.6 %	1.52 (0.71–3.26)	0.28	1.30 (0.60–2.85)	0.51
1 year						
Death	7.5 %	17.4 %	0.39 (0.18–0.84)	0.016	0.37 (0.16–0.84)	0.017
Men	*n* = 541	*n* = 273				
30 day						
Death	3.7 %	5.5 %	0.66 (0.33–1.31)	0.24	0.69 (0.35–1.39)	0.27
Death + nonfatal reinfarction	4.6 %	7.0 %	0.65 (0.35–1.20)	0.17	0.68 (0.37–1.28)	0.23
Death + nonfatal reinfarction + urgent revascularization	5.0 %	8.1 %	0.60 (0.34–1.07)	0.09	0.64 (0.36–1.17)	0.15
Major bleeding requiring transfusion	1.3 %	1.1 %	1.18 (0.30–4.60)	0.81	1.40 (0.35–5.64)	0.64
Intracranial haemorrhage	0 %	0 %	–	–	–	–
Puncture site haematoma	6.7 %	7.3 %	0.90 (0.51–1.59)	0.72	0.94 (0.53–1.68)	0.83
All bleeding	7.6 %	8.1 %	0.94 (0.55–1.61)	0.81	0.98 (0.57–1.71)	0.95
1 year						
Death	5.2 %	8.1 %	0.62 (0.35–1.11)	0.11	0.68 (0.38–1.22)	0.19

Values are presented as percentages and unadjusted and adjusted for propensity score odds ratios (OR) with 95 % confidence intervals (CI)

## Discussion

The main finding of the present study is that among women with STEMI, the early use of abciximab enhances myocardial reperfusion before and after primary PCI, and leads to improved survival.

Women presenting with STEMI are high-risk individuals, with advanced age, and higher prevalence of comorbidities than man [9–11]. In addition, successful reperfusion in women is frequently delayed due to an atypical presentation. Contrary to reports from De Luca et al. [10, 11] and Lansky et al. [9] suggesting no impact of gender on reperfusion success, in our study female patients were less likely to achieve TIMI grade 3 flow and more likely to have angiographic complications after primary PCI than did men. Importantly, female patients with STEMI treated with primary PCI had higher unadjusted 30 day and 1 year mortality. However, that difference in mortality between women and men is related rather to the difference in their risk profile, and female sex did not emerge as an independent predictor of death [9–11]. The observed difference in bleeding was driven principally by the higher rate of puncture site hematomas in female patients. Interestingly, female gender carries an increased risk of local hematomas even in patients treated through the radial approach during primary PCI [12].

GPIIb–IIIa inhibition during primary PCI improves myocardial perfusion and long-term survival [13]. However, in a meta-regression analysis from De Luca et al. [1], the mortality benefit of GPIIb–IIIa inhibitors during primary PCI for STEMI was limited to high-risk patients. Data assessing the impact of gender on the safety and efficacy of GPIIb–IIIa inhibitors during primary PCI are limited. Pooled analysis from EPIC, EPILOG and EPISTENT trials demonstrated no gender difference in protection from major adverse outcomes with GPIIb–IIIa inhibition with abciximab during planned or urgent PCI [14]. Although women had higher rates for both major and minor bleeding events with abciximab compared with men. In contrary, in the ISAR-REACT 2 study higher effectiveness of abciximab used on top of 600 mg clopidogrel loading-dose among men than women was confirmed in patients with non-ST-elevation acute coronary syndrome undergoing PCI [15]. On the other hand, in the CADILLAC study the addition of abciximab during primary PCI for STEMI in female patients significantly reduced 30 day target-vessel revascularization without increasing bleeding risk, especially when women were treated with stent [9]. As confirmed by HORIZONS-AMI study, replacement of combination of unfractionated heparin and GPIIb–IIIa inhibitor with bivalirudin during primary PCI may reduce the rates of major bleeding and net adverse clinical events during 30 day follow-up, and both of these effects are independent of gender [16].

Additional clinical benefit may be achieved in patients with STEMI when GPIIb–IIIa inhibitor (abciximab) is given upstream, before primary PCI [2–6]. However, present analysis confirmed that mortality benefit of early abciximab administration may be limited to patients with a higher baseline risk profile, including female patients. This finding is in line with our previous reports showing that early administration of abciximab before transportation for primary PCI is associated with significantly lower 1 year mortality in high-risk patients (TIMI risk score ≥3), diabetic, and elderly patients (≥65 years of age) [2, 3, 17]. Importantly, upstream use of abciximab was not associated with excessive bleeding risk, including no increase in major bleeding requiring transfusion in comparison to administration during PCI in both women and men. Similarly to our results, in the EGYPT cooperation analysis on patients with STEMI early administration of GPIIb–IIIa inhibitors enhanced patency of the infarct-related artery before and after primary PCI and led to mortality reduction (for patients treated with abciximab) [5]. No interaction between gender and effects of upstream GPIIb–IIIa inhibitors administration on mortality was confirmed. In the recently published MISTRAL study, despite enhancement of early reperfusion before PCI, and reduction of the risk of angiographic complications of PCI no mortality benefit was confirmed for in the ambulance versus in the cathlab use of abciximab [18]. Importantly, the MISTRAL study included rather small and highly selected population with a low-risk profile (Killip class >I in 8 % of patients, diabetes mellitus in 8 %, and overall mortality 1.17 %). Results achieved by women and men in this study have not yet been reported. In the On-TIME-2 study prehospital initiation of another GPIIb–IIIa inhibitor (high-bolus dose of tirofiban) improved ST-segment resolution and clinical outcome after primary PCI, however no difference in mortality between study groups was observed [19]. Importantly, the benefits of tirofiban in terms of reduction of primary efficacy endpoint (the extent of residual ST-segment deviation at 1 h after PCI) were more pronounced in women than men.

In our registry, abciximab was given intravenously. There is growing interest in the intracoronary [20] or intralesion [21] administration of abciximab via dedicated therapeutic perfusion catheter. Local administration of abciximab increases concentrations at the culprit lesion and in the distal vascular bed, and allows to improve the diffusion of abciximab to platelets within flow-limiting thrombi. By potentiating the local anti-inflammatory effects of abciximab, reperfusion injury may be minimized resulting in greater myocardial salvage. However, no clinical benefit of intracoronary versus intravenous administration of abciximab through the guiding catheter during primary PCI was confirmed in the large, randomized study [20]. In the recently reported INFUSE AMI trial local administration of abciximab was associated with significant, however, modest reduction of the infarct size assessed by cardiac magnetic resonance at 30 days after first, anterior wall STEMI in comparison to no abciximab administration [21]. No data comparing early administration of abciximab vs intracoronary administration are available.

New P2Y12 inhibitors–prasugrel [22] and ticagrelor [23] may overcome limitations of clopidogrel in patients with STEMI, as both have shown more potent antiplatelet effect and a higher clinical benefit. Present study suggests that early abciximab administration is still a reasonable strategy for high-risk patients with STEMI, including women when the bleeding risk profile is acceptable, however the relevance of that treatment strategy on top of new oral antiplatelet agents, especially given in a prehospital setting requires further studies.

## Limitations

Several important limitations need to be kept in mind when interpreting the results of this study. The main limitation is the non-randomized nature of the study and the potential of selection bias. Even using propensity score adjustment, we were unable to control all patients, operator and center-related factors influencing an association between early abciximab use and patient outcome. Interpretation of TIMI flow, no-reflow, distal embolization, and ST-segment resolution were limited by the fact that these represent not an independent core laboratory but physicians assessments. Unfortunately, important data on either thrombus grading or myocardial perfusion grading were not available. However, these limitations might not influence the results of the study, as both groups were exposed to confounding factors and were relatively large.

## Conclusions

Early administration of abciximab improved patency of the infarct-related artery before and after PPCI, and led to improved survival in female patients with STEMI.
